# Early Virulence Predictors during the *Candida* Species–*Galleria mellonella* Interaction

**DOI:** 10.3390/jof6030152

**Published:** 2020-08-27

**Authors:** Laura C. García-Carnero, Diana M. Clavijo-Giraldo, Manuela Gómez-Gaviria, Nancy E. Lozoya-Pérez, Alma K. Tamez-Castrellón, Luz A. López-Ramírez, Héctor M. Mora-Montes

**Affiliations:** Departamento de Biología, División de Ciencias Naturales y Exactas, Campus Guanajuato, Universidad de Guanajuato, Noria Alta s/n, col. Noria Alta, C.P., Guanajuato Gto. 36050, Mexico; laura_cgc@hotmail.com (L.C.G.-C.); diamar438@hotmail.com (D.M.C.-G.); manuela.gomezg8@gmail.com (M.G.-G.); nelppat@hotmail.com (N.E.L.-P.); soulk_taca@hotmail.com (A.K.T.-C.); adrianalr@ugto.mx (L.A.L.-R.)

**Keywords:** lactate dehydrogenase, melanin, virulence predictors, invertebrate infection model, hemocytes, phenoloxidase

## Abstract

Fungal infections are a serious and increasing threat for human health, and one of the most frequent etiological agents for systemic mycoses is *Candida* spp. The gold standard to assess *Candida* virulence is the mouse model of systemic candidiasis, a restrictive, expensive, and time-consuming approach; therefore, invertebrate models have been proposed as alternatives. *Galleria mellonella* larvae have several traits that make them good candidates to study the fungal virulence. Here, we showed that a reduction in circulating hemocytes, increased melanin production, phenoloxidase, and lactate dehydrogenase activities were observed at 12 and 24 h postinoculation of highly virulent *Candida* *tropicalis* strains, while minimal changes in these parameters were observed in low-virulent strains. Similarly, the most virulent species *Candida albicans*, *Candida tropicalis*, *Candida auris*, *Candida parapsilosis*, and *Candida orthopsilosis* have led to significant changes in those parameters; while the low virulent species *Candida guilliermondii*, *Candida krusei*, and *Candida metapsilosis* induced modest variations in these immunological and cytotoxicity parameters. Since changes in circulating hemocytes, melanin production, phenoloxidase and lactate dehydrogenase activities showed a correlation with the larval median survival rates at 12 and 24 h postinoculation, we proposed them as candidates for early virulence predictors in *G. mellonella*.

## 1. Introduction

The human fungal infections are caused by a reduced number of etiological agents when compared to the vast range of bacteria that can cause human diseases; nevertheless, the mycoses are a serious threat for human beings, causing morbidity and mortality rates that surpass the one million affected persons per year [1,2]. The most frequent systemic and deep-seated mycoses are cryptococcosis, aspergillosis, and candidemia, caused by *Cryptococcus* spp., *Aspergillus fumigatus*, and *Candida* spp., respectively [1]. *Candida albicans* is still the leading species in most of the candidiasis and candidemia cases, although other species like *Candida tropicalis*, *Candida auris*, *Candida parapsilosis*, *Candida orthopsilosis*, *Candida metapsilosis*, *Candida glabrata*, *Candida guilliermondii*, and *Candida krusei* are relevant etiological agents of these infections [1,3,4,5,6,7]. *Candida* spp. belongs to the microbiota of the mucosal surfaces and skin, colonizing the gastrointestinal and genitourinary tracts of healthy individuals and establishing commensalism with the host [8]. However, changes in the microbiota population, disruption of natural barriers that contain tissues and organs, or affection of the host immunological status could lead to the establishment of the pathogenic process that can cause life-threatening infections [9,10].

The conventional strategy to study the virulence factors and determinants that are behind the aggressivity and resilience of the fungal pathogen, when invading the host tissues, includes the assessment of the fungal ability to colonize organs and to kill laboratory animals. Despite several mammalians having been tested as candidates to analyze fungal virulence, mice are the most standardized animal model for experimental disseminated candidiasis [11,12]. In this model, the *Candida* yeast cells are injected in the lateral tail vein, and this allows spreading to the body via the blood system. Kidneys, spleen, liver, and brain are usually the organs where high fungal loads are found and the malfunctioning of these eventually leads to animal death [12]. However, the requirement of specialized and extensive facilities for animal housing and experimentation [13], and the increased concern about animal wellbeing and regulations for their inclusion in experimentation are among the main limitations to keep this model as the gold standard to assess fungal virulence [14].

Invertebrates models have raised as a popular alternative to assess fungal virulence, because of the minimal requirements for housing, breeding, and the possibility to include large numbers of individuals in the experimental population, proving statistical strength. The most popular systems already set up to study fungal virulence include *Acanthamoeba castellanii*, *Dictyostelium discoideum*, *Caenorhabditis elegans*, *Drosophila melanogaster*, *Tenebrio molitor*, *Bombyx mori,* and *Galleria mellonella* [15,16,17]. *G. mellonella* larvae have thermotolerance and can grow at 37 °C, which represents an advantage to study *Candida* spp. thermoadaptation and temperature related-factors, including dimorphism [15,18,19,20,21,22], and the *G. mellonella* hemocytes, which are immunological cells found in the hemolymph, can perform fungal phagocytosis like mammalian macrophages, and even *Candida* cells can use similar strategies to evade killing in both hemocytes and macrophages [15,23,24]. Moreover, these larvae have demonstrated to be a proper host to evaluate the effect of antifungal drugs and new compounds with antifungal properties on the host–fungus interaction [25,26,27]. Melanogenesis is an important immune response dependent on the phenoloxidase activity, a humoral protein that activates this mechanism in invertebrates by converting phenols to quinones, which finally polymerizes to form melanin [28]. This pigment plays several roles in the immune response, mainly by being deposited around the pathogen or the damaged area. For this, hemocytes gather around the invading pathogen and release chemoattractants, to form a multicellular plasmocytes wall that fills up with melanin, generating a capsule that limits the pathogen, avoiding its growth, spread, and causing death by starvation [28,29]. In addition, intermediates from the melanin pathway can directly kill the pathogen [29]. The *G. mellonella* cells contain the soluble cytoplasmic lactate dehydrogenase (LDH) [18], which like in the mammalian cells is rapidly released when the plasma membrane permeability is compromised [30]. The measuring of this enzyme activity is a common method used to determine cytotoxicity and cellular damage in both mammalian and insect cells [18,30,31].

The most frequent parameters evaluated during the *Candida*–*G. mellonella* interaction are the time taken to kill the animal population, the fungal burden recovered from infected animals, and the expression of genes related to humoral immune factors, such as antimicrobial peptides [18,19,20,21,22,32]. To generate killing curves, an observation period of two weeks is usually required, in particular when the fungal strains show virulence attenuation [18,19,20,21,22]. Furthermore, reductions in circulating hemocytes, melanin production, and phenoloxidase activity have been observed in infected larvae [33].

Here, to explore for early virulence predictors during the *C. tropicalis*–*G. mellonella* interaction, we found that reduction in the circulating hemocytes, increment in melanin production, phenoloxidase activity, and release of lactate dehydrogenase were parameters that correlated with the *C. tropicalis* virulence. In addition, we assessed the usefulness of these parameters as early predictors of other *Candida* species virulence.

## 2. Materials and Methods

### 2.1. Strains and Culture Conditions

The strains used in this study are listed in Table 1. Cells were maintained and propagated at 28 °C in YPD medium (2% (*w*/*v*) bacteriological peptone, 1% (*w*/*v*) yeast extract, and 2% (*w*/*v*) glucose). Liquid cultures were incubated in orbital shakers at 200 rpm. For solid cultures, the medium was added with 2% (*w*/*v*) agar.

### 2.2. Galleria mellonella Survival Assays

The *G. mellonella* larvae were provided from a previously established colony kept in our laboratory [16]. Larvae were fed ad libitum with a diet based on corn bran and honey (1 kg corn bran, 150 g rice meal, 250 mL bee honey, and 70 mL glycerin). Larvae with a length of 1.2–1.5 cm and no visual signs of injuries or melanization were used for the inoculation experiments. The assays were performed as previously described [22]. The last left pro-leg was sanitized with 70% (*v*/*v*) ethanol and 10 µL of PBS containing 2 × 10^7^ yeast cells were injected in this area, using a Hamilton syringe equipped with a 26-gauge needle. Since the mutant strains tend to form cell aggregates, the cell suspensions were passed through a syringe with a 32-gauge needle before inoculated into the animals with a Hamilton syringe equipped with a 26-gauge needle. Larvae from the same experimental group were kept at 37 °C in Petri dishes containing chopped apple for animal hydration, and survival monitored daily. The silk on the animal surface was removed to delay the pupa formation. Both loss of irritability and the presence of extensive body melanization were taken as signs of animal death. Each experimental group contained 30 larvae, and one animal group injected with PBS was included as a control.

### 2.3. Analysis of Hemocyte Levels, Melanin Production, and Phenoloxidase Activity

To collect hemolymph, groups of 10 animals were inoculated as described in the previous section and incubated at 37 °C for 2, 6, 12, or 24 h before decapitation with a sterile scalpel. Thirty µL of hemolymph were recovered from each larva and mixed with 150 µL of anticoagulant solution (93 mM NaCl, 100 mM glucose, 30 mM trisodium citrate, 26 mM citric acid, 10 mM Na_2_EDTA, and 0.1 mM phenylthiourea, pH 4.6). The preparations were mixed gently, stored on ice to minimize cell clumping, and processed the same day to avoid result bias because of sample storage [39]. Hemocytes were quantified in a hemocytometer, as described [40]. Melanin production was assessed by measuring the absorbance at 405 nm of hemolymph, as reported [27]. Briefly, the pooled hemolymph from animals belonging to the same group was analyzed by measuring the absorbance at 405 nm using a MultiskanTM FC microplate photometer (Thermo Fisher Scientific, Waltham, MA, USA). The hemolymph from non-infected larvae was used as a control, and the readings generated with these samples were used for background correction. Phenoloxidase activity was quantified as reported [41]. Briefly, the hemolymph was centrifuged at 20,000× *g* for 10 min, the supernatant saved and used to quantify protein concentration with the Pierce BCA Protein Assay (Thermo Fisher Scientific, Waltham, MA, USA). Reactions were performed in 96-well microplates with a final volume of 200 µL. Aliquots containing 100 µg protein were mixed with 20 mM 3,4-DihydroxyDL-phenylalanine (Sigma-Aldrich, St. Louis, Missouri, USA), the absorbance at 490 nm read in a MultiskanTM FC microplate photometer (Thermo Fisher Scientific, Waltham, MA, USA), the reaction incubated for 30 min at 37 °C, and the optical density at the same wavelength measured again. Enzyme activity was defined as the change in the absorbance and normalized to one minute and one µg protein [39].

To determine the released LDH, hemolymph samples were analyzed with the Pierce LDH Cytotoxicity Assay (Thermo Fisher Scientific, Waltham, MA, USA) [18]. The LDH activity of fresh cell homogenates was regarded as the 100% cytotoxicity, and samples from non-infected larvae were used as controls. The absorbances at 490 nm and 680 nm were obtained using a MultiskanTM FC microplate photometer (Thermo Fisher Scientific, Waltham, MA, USA).

### 2.4. Ethics Statement

The use of animals in this study was approved by the Ethics Committee of Universidad de Guanajuato (permission CIBIUG-P12-2018).

### 2.5. Statistical Analysis

Statistical analysis was performed using the GraphPad Prism 6 software. Survival experiments were performed with a total of 30 larvae per group, data plotted in Kaplan–Meier survival curves, and analyzed using the log-rank test. Other results were analyzed with the Mann–Whitney U test. To establish a correlation between animal mortality and hemocyte, melanin, phenoloxidase, or cytotoxicity, the Spearman nonparametric correlation was used. The statistical significance in all cases was set at *p* < 0.05. All data are represented as mean and standard deviation (SD).

## 3. Results

### 3.1. The Virulence Defect of C. tropicalis mnn4Δ, och1Δ, and pmr1Δ Null Mutants in G. mellonella Larvae

**Figure 1:**
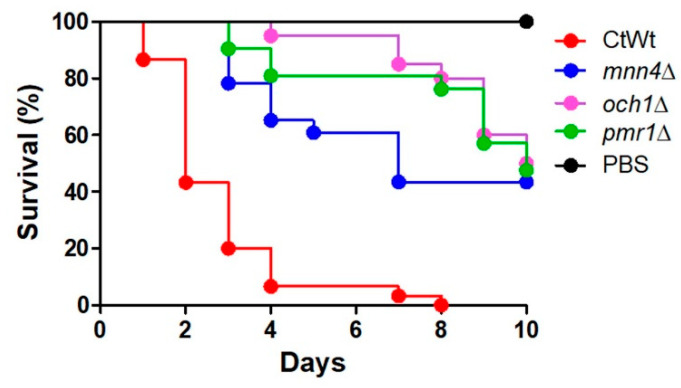
Mortality curves of *Galleria mellonella* larvae infected with *Candida tropicalis* WT, *mnn4*Δ, *pmr1*Δ, or *och1*Δ null mutant strains. Each animal group contained 30 larvae (10 larvae for each experiment) and animals were infected with 2 × 10^7^ yeast cells. Animal death was defined by extensive body melanization and loss of irritability, parameters that were monitored daily. PBS, control group injected only with PBS. Strains used are MYA-3404 (WT), HMY175 (*mnn4*Δ), HMY181 (*och1*Δ), and HMY207 (*pmr1*Δ). The three null mutant strains showed a significant difference in the ability to kill larvae when compared to the WT strain (*p <* 0.05 in all cases).

We have recently demonstrated that disruption of the protein glycosylation pathways affected *C. tropicalis* virulence in *G. mellonella* larvae [19,20]. The *MNN4* disruption reduced the *C. tropicalis* ability to kill animals but the fungal burden recovered from the hemolymph was similar to that found in the animals infected with the wild-type (WT) strain [20]; however, the disruption of either *OCH1* or *PMR1* negatively affected both the number of animals killed and the fungal burdens associated to the null mutant strains [19]. Due to this differential behavior of *C. tropicalis* mutant strains when interacting with *G. mellonella* larvae, we used them to explore for early indicators of their virulence in this animal model. We first confirmed the virulence attenuation of these strains, defined by the inability to kill the animal population during the observation period. The mortality curve associated with the WT strain (ATCC MYA-3404) showed that all animals were killed at day 8 postinoculation with a median survival of two days (Figure 1), and as expected, the three null mutants under analysis were capable of killing only 50% of the animal population, only when the observation period was extended to 10 days (Figure 1). The median survival was 7 days for animals infected with the *mnn4*Δ null mutant and 10 days for animals infected with either *och1*Δ or *pmr1*Δ mutant strains. Even though the *mnn4*Δ null mutant strain seemed to kill faster the animal population than the other mutant strains, these differences were not statistically significant (*p* = 0.27 when *mnn4*Δ and *och1*Δ strains were compared; *p* = 0.45 when *mnn4*Δ and *pmr1*Δ were compared). Therefore, these data confirmed the virulence attenuation of the three null mutant strains under study.

**Figure 2:**
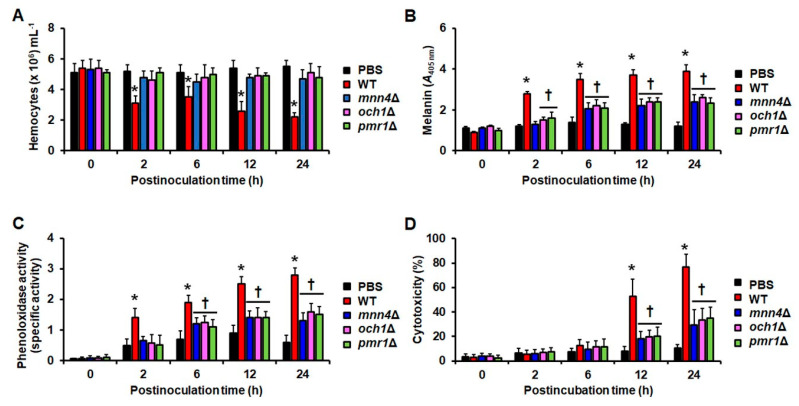
Quantification of hemocytes, melanin, phenoloxidase activity, and cytotoxicity in *Galleria mellonella* larvae infected with *Candida tropicalis* WT, *mnn4*Δ, *pmr1*Δ, or *och1*Δ null mutant strains. Groups of 10 animals were infected with 2 × 10^7^ yeast cells, incubated for the indicated time at 37 °C, animals were decapitated, hemolymph collected and used to quantify the hemocytes concentration (**A**), melanin production (**B**), phenoloxidase activity (**C**), or cytotoxicity (**D**). In C, the specific activity was defined as the change in the absorbance at 490 nm per one minute per one µg protein. In D, cytotoxicity was measured as the lactate dehydrogenase activity in the cell-free hemolymph. The 100% cytotoxicity corresponds to the enzyme activity quantified from lysed hemocytes. Strains used are MYA-3404 (WT), HMY175 (*mnn4*Δ), HMY181 (*och1*Δ), and HMY207 (*pmr1*Δ). Data are plotted as means ± standard deviation of three independent experiments performed by duplicated. * *p* < 0.05 when compared to the other values gathered at the same postinoculation time. † *p <* 0.05 when compared to the control condition at the same postinoculation time.

The first parameters we assessed as early predictors of infection were related to the *G. mellonella* immune response. As mentioned, hemocytes are immune cells that are found in the insect hemolymph and are in charge of pathogen phagocytosis [40]. We quantified the hemocyte number immediately after injection of fungal cells (time 0), or after 2, 6, 12, or 24 h of cell inoculation. We restricted our observation period to the first 24 h of interaction because at this time point only 15% of the animal population infected with the WT strain died, longer interaction times were associated with increased numbers of killed animals (Figure 1), and we were looking for early predictors of the interaction outcome. The control group, injected only with PBS, did not show changes in the hemocyte counts during the observation period (Figure 2A), but when the WT strain-larva interaction was analyzed, a significant reduction in the hemocyte number was observed at 2 h postinoculation and the following observation points (Figure 2A). The three null mutants under study showed similar hemocyte countings during all the analyzed points, and these figures were not significantly different from those obtained with the control group but were higher than those found in animals infected with the WT strain (Figure 2A). These data suggested that the fast animal killing of the WT control strain could be associated with low hemocyte countings during the first day of the pathogen–host interaction. Next, we compared the presence of melanin in the cell-free fraction of the hemocele of larva interacting with fungal cells. Melanin production is one of the humoral strategies that *G. mellonella* and other insects have evolved to encapsulate and control the pathogen dissemination when entering into the hemocele [41]. The production of this pigment was similar in all the samples collected from the control group, with a discrete peak at 6 h postinoculation that was not significantly different when compared to the other samples (*p >* 0.1; Figure 2B). In animals infected with the WT control cells, melanin production was significantly higher after 2, 6, 12, and 24 h postinoculation, when compared with the samples from the control group (Figure 2B). The highest melanin production in these animals was observed at 6 h postinoculation and even though the production of this pigment seemed to increase at 24 h postinoculation, this was not statistically significant to the level observed at 6 or 12 h (Figure 2B). At 2 h postinoculation, melanin production in the infected larvae with either the *och1*Δ or *pmr1*Δ null mutants was higher than in the control group, while the pigment production by animals infected with the *mnn4*Δ null mutant was similar to those levels found in the control group (Figure 2B). Upon 6 h of interaction, melanin production in animals infected with any of the three null mutants was higher to the levels found in the control group and did not significantly change at 12 or 24 h postinoculation (*p >* 0.1 for both time points). However, in all the cases, melanin production in animals infected with the null mutant cells was lower than the pigment produced by animals infected with the WT control strain (Figure 2B). When phenoloxidase activity [41], a key enzyme involved in the insect melanin synthesis, was measured, there was an increment of enzyme activity at 2 h postinoculation in the control group, and although this activity increased at 12 h postinoculation, this was not significant (*p >* 0.1; Figure 2C). For the case of animals infected with the WT control cells, there was a time-dependent increment in the enzyme activity, with the maximal activity reached at 24 h postinoculation (Figure 2C). For the three null mutant strains analyzed, phenoloxidase activity at 2 h postinoculation was similar among them and to those levels observed in the control group, but a significant increment was observed at 6, 12, and 24 h postinoculation; although this was not dependent on the interaction time (Figure 2C). Collectively, these data indicated that the *mnn4*Δ, *och1*Δ, and *pmr1*Δ null mutants induced low melanin production, and this correlated with lower phenoloxidase activity in animals when compared with the group infected with WT control cells.

It has been previously reported that LDH released into the hemolymph of larva infected with either bacteria or fungal cells is a useful parameter to assess pathogen-associated cytotoxicity [18,42]. Thus, we next assessed whether this cytotoxicity could be present during the early times of the host–*C. tropicalis* interaction. The cytotoxicity found in animals injected with PBS was minimal at 2 h postinoculation and it reached about 10% cytotoxicity at 24 h postinoculation (Figure 2D). For the larvae infected with the WT strain, cytotoxicity was only evident at 12 and 24 h postinoculation points, while the LDH level found at 0, 2, and 6 h postinoculation was similar to that found in the control group (Figure 2D). The three null mutants generated a similar cytotoxicity degree, which was also evident at 12 and 24 h postinoculation but was significantly lower than that found in the animals infected with the WT control cells (Figure 2D). These data suggested a correlation between low virulence and low cytotoxicity for the three null mutants.

### 3.2. Early Virulence Predictors in Larva Infected with C. tropicalis Clinical Isolates

**Figure 3:**
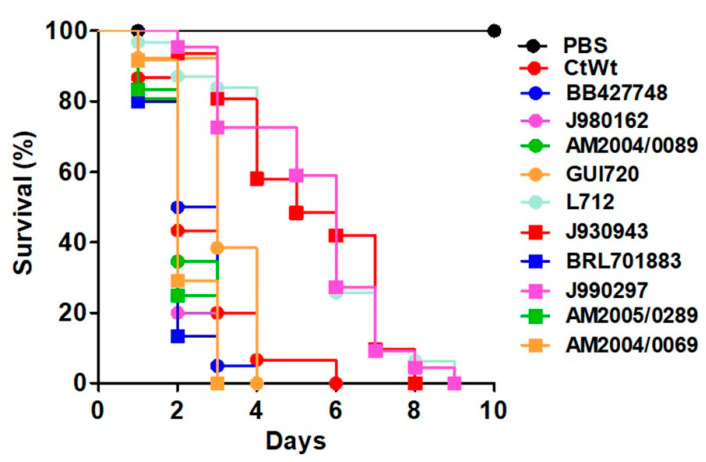
Mortality curves of *Galleria mellonella* larvae infected with different *Candida tropicalis* clinical isolates. Each animal group contained 30 larvae (10 larvae for each experiment) and animals were infected with 2 × 10^7^ yeast cells. Animal death was defined by extensive body melanization and loss of irritability, parameters that were monitored daily. PBS, control group injected only with PBS. WT, strain MYA-3404. The mortality in animals infected with strains L712, J930943, and J990297 was significantly different from that associated with the other fungal strains (*p <* 0.05 in all cases).

**Figure 4:**
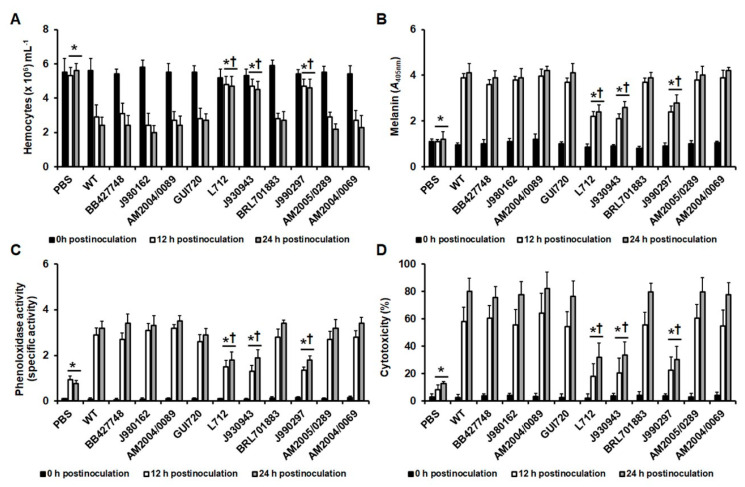
Quantification of hemocytes, melanin, phenoloxidase activity, and cytotoxicity in *Galleria mellonella* larvae infected with *Candida tropicalis* clinical isolates. Groups of 10 animals were infected with 2 × 10^7^ yeast cells, incubated for the indicated time at 37 °C, animals were decapitated, hemolymph collected and used to quantify the hemocytes concentration (**A**), melanin production (**B**), phenoloxidase activity (**C**), or cytotoxicity (**D**). In C, the specific activity was defined as the change in the absorbance at 490 nm per one minute per one µg protein. In D, cytotoxicity was measured as the lactate dehydrogenase activity in the cell-free hemolymph. The 100% cytotoxicity corresponds to the enzyme activity quantified from lysed hemocytes. WT, strain MYA-3404. Data are plotted as means ± standard deviation of three independent experiments performed by duplicated. * *p <* 0.05 when compared to the values gathered at the same postinoculation time in animals infected with the WT strain. † *p <* 0.05 when compared to the control condition at the same postinoculation time.

The parameters analyzed in the larva infected with the *C. tropicalis* WT strain and the *mnn4*Δ, *och1*Δ, and *pmr1*Δ null mutants indicated that virulence attenuation found in the mutant strains, defined as decreased ability to kill the animal population at a similar rate as the WT strain, can be associated to minimal changes in hemocyte countings, to low melanin production, low phenoloxidase activity, and reduced cytotoxicity. The three immunological parameters tested here suffered changes since 2 h postinoculation and the trend continued during the first 24 h after the pathogen inoculation. However, LDH release was evident only at 12 and 24 h postinoculation, in the four analyzed strains. We hypothesized that the changes in these parameters at 12 and 24 h postinoculation times could be correlated with the mortality of animals infected with different *C. tropicalis* clinical isolates. When these strains were injected into *G. mellonella* larvae we could identify two different groups. Strains BB427748, J980162, AM2004/0089; GUI720; BRL701883, AM20050289, and AM2004/0069 killed the animal population at a similar rate to the WT strain, with a median survival of 2.2 ± 0.4 days, which was shorter to that found for strains L712, J930943, and J990297 that have a median survival of 5.3 ± 0.6 days (Figure 3). The colony fungal units recovered from the infected animals were similar for all the strains analyzed. When the hemocyte levels in animals infected with these clinical isolates were analyzed, we found that strains BB427748, J980162, AM2004/0089; GUI720; BRL701883, AM20050289, and AM2004/0069 stimulated the reduction of these cells in the hemolymph, in a similar manner than the WT strain at both 12 and 24 h postinoculation (Figure 4A). On the contrary, strains L712, J930943, and J990297 stimulated the reduction in the circulating levels of these cells but this was not as pronounced as those cell values associated with the other strains under analysis (Figure 4A). This reduction in hemocyte countings associated with the infection with WT, BB427748, J980162, AM2004/0089; GUI720; BRL701883, AM20050289, or AM2004/0069 strains showed a good positive correlation with the median survivals (r = 0.807, 0.783, 0.707, 0.749, 0.786, 0.807, 0.858, and 0.764, respectively). Melanin production, phenoloxidase activity and cytotoxicity in animals infected with strains L712, J930943, and J990297 were significantly different with those values observed in animals injected with PBS but were significantly lower when compared with the levels observed in larvae infected with strains BB427748, J980162, AM2004/0089; GUI720; BRL701883, AM20050289, or AM2004/0069, which again, behaved like the WT strain at both observation times (12 and 24 h postinoculation; Figure 4B–D). The low melanin production, phenoloxidase activity, and cytotoxicity in animals infected with strains L712, J930943, or J990297 showed a negative correlation with the median survival of infected animals (for melanin production r = −0.756, −0.793, and −0.868, respectively; for phenoloxidase activity r = −0.730, −0.705, and −0.893; for cytotoxicity r = −0.804, 0.773, and −0.839, respectively). Collectively, these data suggest that high hemocyte countings into the hemolymph and low melanin, phenoloxidase activity, and cytotoxicity correlated with low larval killing rate.

### 3.3. Early Virulence Predictors in Larva Infected with Candida Species

**Figure 5:**
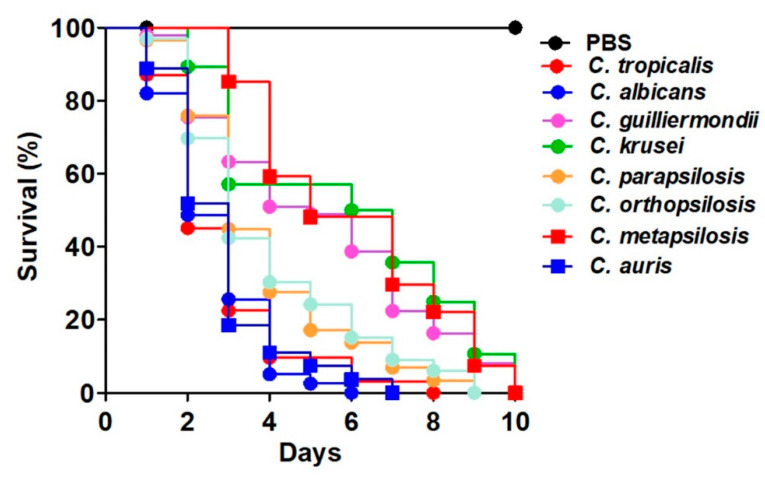
Mortality curves of *Galleria mellonella* larvae infected with different *Candida* species. Each animal group contained 30 larvae (10 larvae for each experiment) and animals were infected with 2 × 10^7^ yeast cells. Animal death was defined by extensive body melanization and loss of irritability, parameters that were monitored daily. PBS, control group injected only with PBS. *Candida albicans*, *Candida tropicalis*, and *Candida auris* were the fastest species in killing the larvae population; while the slowest species in killing the animal population were *Candida metapsilosis*, *Candida guilliermondii*, and *Candida krusei*. *Candida parapsilosis* and *Candida orthopsilosis* conformed the third group in between the fastest and lowest species groups.

Next, to explore whether these early virulence predictors identified in animals infected with *C. tropicalis* could also be informative during the *Candida* spp.–*G. mellonella* interaction, we first determined the mortality in larvae infected with *Candida albicans*, *Candida parapsilosis*, *Candida orthopsilosis*, *Candida metapsilosis*, *Candida auris*, *Candida guilliermondii*, or *Candida krusei*. The killing curves indicated that *C. albicans*, *C. tropicalis*, and *C. auris* were the fastest species to kill the animal population, with a median survival of 2.3 ± 0.6 days and with no significant differences in their killing curves (*p* = 0.35), followed by animals infected with *C. parapsilosis* and *C. orthopsilosis,* which showed a median survival of 3.5 ± 0.7 days (*p <* 0.05 when compared with the killing curves of species belonging to the fastest group; *p* = 0.29 when compared the curves of animals infected with *C. parapsilosis* and *C. orthopsilosis*); while *C. metapsilosis*, *C. krusei*, and *C. guilliermondii* were the strains that showed the lowest killing rate of *G. mellonella* larva, as these had a median survival of 5.5 ± 0.9 days (*p <* 0.05 when compared with the curves generated with *C. albicans*, *C. tropicalis*, *C. auris; C. orthopsilosis, C. parapsilosis*; *C. metapsilosis*, *C. krusei*, or *C. guilliermondii*; Figure 5).

**Figure 6:**
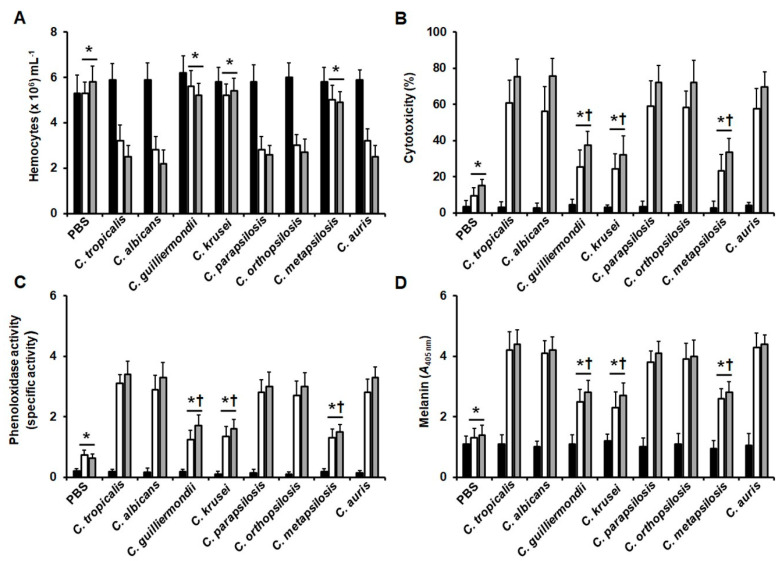
Quantification of hemocytes, melanin, phenoloxidase activity, and cytotoxicity in *Galleria mellonella* larvae infected with different *Candida* species. Groups of 10 animals were infected with 2 × 10^7^ yeast cells from the different species under study, incubated for the indicated time at 37 °C, animals were decapitated, hemolymph collected and used to quantify the hemocytes concentration (**A**), cytotoxicity (**B**), phenoloxidase activity (**C**), or melanin production (**D**). In B, cytotoxicity was measured as the lactate dehydrogenase activity in the cell-free hemolymph and the 100% cytotoxicity corresponds to the enzyme activity quantified from lysed hemocytes. In C, the specific activity was defined as the change in the absorbance at 490 nm per one minute per one µg protein. Closed, open, and grey bars correspond to measurements at 0 h, 12 h, and 24 h postinoculation, respectively. Data are plotted as means ± standard deviation of three independent experiments performed by duplicated. * *p <* 0.05 when compared to the values gathered at the same postinoculation time in animals infected with *C. tropicalis*. † *p <* 0.05 when compared to the control condition at the same postinoculation time.

When the immunological parameters were quantified in the infected animals, we found that as showed in the previous sections, the hemocytes recovered from the hemolymph of animals infected with *C. tropicalis* were drastically reduced at 12 and 24 h postinoculation, and a similar trend was observed in animals infected with *C. albicans*, *C. parapsilosis*, *C. orthopsilosis,* or *C. auris* (Figure 6A). On the contrary, the hemocyte levels in animals infected with *C. guilliermondii*, *C. krusei,* or *C. metapsilosis* were significantly higher in both time periods, and no statistical significance was observed when compared among them (Figure 6A; *p >* 0.05 in all cases). For all the strains under analysis, the difference between the cell quantified at 12 and 24 h was not significant (*p >* 0.05). The reduction in the hemocyte levels at 12 and 24 h postinoculation showed a positive correlation with the median survival of animals infected with *C. tropicalis*, *C. albicans*, *C. parapsilosis*, *C. orthopsilosis*, or *C. auris* (r = 0.859, 0.835, 0.798, 0.874, and 0.843, respectively). For the case of melanin production, phenoloxidase activity, and cytotoxicity, the three parameters were higher at 12 and 24 h postinoculation when compared with the values observed at time 0 h, and these levels were significantly lower in animals infected with *C. guilliermondii*, *C. krusei*, or *C. metapsilosis* (Figure 6B–D). The melanin production, phenoloxidase activity, and cytotoxicity in animals infected with *C. tropicalis*, *C. albicans*, *C. parapsilosis*, *C. orthopsilosis*, or *C. auris* were similar among them (Figure 6B–D). In all cases, even though there was a trend in the values to increment during the interaction time, this was not significant (*p >* 0.05 in all cases). The low melanin production, phenoloxidase activity, and cytotoxicity in animals infected with *C. guilliermondii*, *C. krusei*, or *C. metapsilosis* showed a negative correlation with the median survivals (for melanin production, r = −0.842, −0.754, and −0.801; for phenoloxidase activity, r = −0.795, −0.801, and −0.824; for cytotoxicity, r = −0.794, −0.762, and −0.854, respectively).

## 4. Discussion

The mouse model of disseminated candidiasis is still regarded as the gold standard to assess *Candida* spp. virulence. A conventional analysis of mortality in animals infected includes an observation period for at least four weeks [11,43], representing an expensive and time-consuming task, which exposes the animals to long times of illness and suffering [43]. Therefore, early predictors of the host–pathogen interaction have been investigated in the murine model. Highly virulent *C. albicans* strains showed high levels of cell infiltrates in kidneys, high fungal burdens in these organs, and increased production of the immune mediators interleukin (IL)-1β, monocyte chemoattractant protein-1, chemoattractant KC, IL-6, granulocyte colony-stimulating factor, tumor necrosis factor, and macrophage inflammatory proteins 2 and 1β at 24 and 48 h postinoculation, respectively [44]. The chemoattractant KC, IL-6, and macrophage inflammatory protein-1β production at 12 h postinoculation were considered as the earliest parameters that correlated with fungal virulence [44]. In addition, loss of body weight, in particular, a 20% reduction during the first four days postinoculation was proposed as a predictor of animal death [45].

In *G. mellonella,* predictors of the host–fungus interaction have been also proposed. It was reported that circulating hemocytes showed a significant reduction in counting after 48 h of inoculation with different highly-virulent *C. albicans* strains; this observation was also reported in animals infected with either *C. parapsilosis* or *C. tropicalis* [23]. On the contrary, no reduction in hemocyte numbers was observed in animals infected with the low-virulent species *C. krusei* [23]. The results reported here confirm these observations and indicated this reduction in circulating hemocytes occurs since the 12 h postinoculation and also in the high-virulent strains *C. auris* and *C. orthopsilosis*. In the same line, the low-virulent strains *C. guilliermondii* and *C. metapsilosis* [46] did not show this reduction in immune cells. It was reported that hemocytes in the hemolymph reduced their numbers at 3 h postinoculation with *C. tropicalis*, but this reduction was not correlated with the virulence of the fungal strain [24]. Despite the mechanism behind this observation is not well characterized, it has been suggested that hemocyte reduction occurs because of the formation of hemocytes–microorganisms clumps [47]. In addition, for the case of fungal species that form true hyphae or pseudohyphae, it has been suggested that similar to the phenomenon described in mammalian macrophages [48], upon phagocytosis by hemocytes, the transition to hypha can generate fungal cells protruding from the immune cells, piercing the cell membrane, and promoting lysis, negatively affecting the cell concentration in the hemolymph [24]. Our results with *C. tropicalis* clearly showed that hemocyte levels were reduced since the 2 h postinoculation, but it remains to be established whether the changes observed in this earlier interaction point could be correlated with median survivals in all the *Candida* species under analysis.

Melanin is synthesized in response to mechanical damage and as a defense mechanism when invading particles or microorganisms enter into the hemocele, being a key process in sclerotization, wound healing, and in defense reactions [49]. This pigment is usually associated with the formation of nodules that reduce pathogen replication [50]. Despite melanin production has been already reported to increment in animals infected with *C. krusei*, *C. albicans*, *C. tropicalis*, *C. glabrata*, or *C. parapsilosis* [27,51,52], the comparative ability of fungal species to stimulate melanin production has not been reported previously, nor the ability of other fungal species to stimulate the production of this pigment, nor the correlation with the median survival time of infected animals. The results reported here support the use of this humoral component of insect immunity as an early predictor of the outcome of the *Candida*–*G. mellonella* interaction, and although other humoral components of the immunity have been explored as early predictors of such interaction [32], there is a significant practical advantage on the measurement of melanin over antimicrobial peptides, as the former relies on spectrophotometrical quantifications.

Few studies have dealt with the role of phenoloxidase during the *G. mellonella* infection with *Candida* spp. This enzyme catalyzes the synthesis of melanin through phenol oxidation to quinones that subsequently polymerize via non-enzymatically reactions, forming melanin [49]. It has been recently demonstrated that phenoloxidase activity increments at 7 and 24 h postinoculation when a non-lethal *C. albicans* inoculum is injected to the animals; however, this activity is strongly repressed when a lethal dose of yeast cells is administered to animals [53]. These data contrast with the observations reported here, as a lethal dose of *C. albicans* induced accumulation of the enzyme activity in the first 24 h postinoculation. One possible explanation to conceal this discrepancy is that the *C. albicans* strains used in both studies are different: here we used the strain SC5314, while in the study above mentioned the strain ATCC 10231 was used for interaction with *G. mellonella* larvae [53]. It has been described that mouse–*C. albicans* interaction is strain-specific, as some immune effectors are stimulated in a strain-specific manner [54]. A similar observation could explain the differential effect of both *C. albicans* strains on the ability to stimulate or repress the phenoloxidase activity. Alternatively, the difference could rely on the experimental setting, as we performed the larva–fungus interaction at 37 °C; whereas in the previous study, these organism–organism interactions were carried out at 28 °C, a restrictive temperature to undergo fungal dimorphism and to express temperature-dependent virulence factors. Because of the roles of both phenoloxidase activity and melanin production in the insect defense mechanisms, it seems contradictory that the high production of both the pigment and enzyme activity was observed in larvae with short median survival times. It is possible to hypothesize that the strong activation of these humoral effectors involves resources reassignment to this humoral response, with detrimental effects on the insect fitness. In addition, it has been suggested that phenoloxidase activity, and therefore melanization, could be activated by recognition of cell wall β-glucan [33], and the exposure of this polysaccharide can vary depending on the fungal species analyzed [3,4,21,22].

The LDH release as a predictor of cytotoxicity in *G. mellonella* has been poorly studied [18,42,55]; however, the results showed here underscore that its quantification is not only a good marker for cytotoxicity but also is a proper early predictor of the outcome between *Candida* spp. and *G. mellonella* larvae.

The *C. tropicalis* strains analyzed here could be classified into two groups, one that killed the larvae as fast as the WT strain and a second with strains that killed the animal population slower. A similar observation has been previously reported, where 53 strains were classified as low (24.5%), moderate (54.7%), and high (20.8%) virulent, showing strain-specific variations in the ability to kill *G. mellonella* larvae [51]. This variation has been also reported for different *C. tropicalis* strains used in the murine model of systemic candidemia [56]. The expression of traditional virulence factors, such as adhesion and secretion of hydrolytic enzymes varies among *C. tropicalis* clinical and environmental isolates [57,58], and this could partially explain the behavior of the strains analyzed here when inoculated within the larvae. In addition, multilocus sequence typing analyses have demonstrated that isolates from this species can be grouped in at least three clades [34], which underscore genome plasticity that could be behind these differences in virulence.

It has been previously reported that *G. mellonella* larvae are a suitable model to study the virulence of *C. albicans*, *C. auris*, *C. tropicalis*, *C. parapsilosis*, *C. orthopsilosis*, *C. metapsilosis*, and *C. krusei* [24,51,59]. The results reported here are in agreement with previously published studies, which reported that *C. albicans*, *C. auris*, *C. tropicalis*, *C. parapsilosis*, and *C. orthopsilosis* are grouped among the most virulent species [46,51], and *C. krusei* and *C. metapsilosis* among the less virulent species [27,46,51,59]. It has to be mentioned though that the killing curves with the different *Candida* species generated three separated groups: the species with the fastest ability to kill the animal population, the slowest group, and the intermediate one. The results using the early virulence predictors could not discriminate between the fastest and the intermediate group to kill the larvae population, an observation that has to be taken into account when analyzing other fungal strains or species. Finally, since the mouse–*C. albicans* interaction is strain-specific [54], and this observation is likely to occur in the *G. mellonella* model, one limitation that we have to acknowledge in our study is that we did not include several isolates of most of the *Candida* species under study, and the reported correlations require further confirmation using other fungal strains.

In conclusion, here we reported that reduction in hemocyte countings, increment in melanin production, phenoloxidase activity, and release of LDH are parameters that correlate with the median survival times of *G. mellonella* larvae infected with *Candida* spp., and can be used as early virulence predictors.

## Figures and Tables

**Table 1:** *Candida* spp. strains used in this work.

Strain	Organism	Genotype	Reference
ATCC MYA-3404	*Candida tropicalis*	Wild-type	ATCC
HMY175	*Candida tropicalis*	As ATCC MYA-3404, but *mnn4*Δ::*sat1*/*mnn4*Δ::*sat1*	[20]
HMY181	*Candida tropicalis*	As ATCC MYA-3404, but *och1*Δ::*sat1*/*och1*Δ::*sat1*	[19]
HMY207	*Candida tropicalis*	As ATCC MYA-3404, but *pmr1*Δ::*sat1*/*pmr1*Δ::*sat1*	[19]
BB427748	*Candida tropicalis*	Wild-type	[34]
J980162	*Candida tropicalis*	Wild-type	[34]
AM2004/0089	*Candida tropicalis*	Wild-type	[34]
GUI720	*Candida tropicalis*	Wild-type	[34]
L712	*Candida tropicalis*	Wild-type	[34]
J930943	*Candida tropicalis*	Wild-type	[34]
BRL701883	*Candida tropicalis*	Wild-type	[34]
J990297	*Candida tropicalis*	Wild-type	[34]
AM2005/0289	*Candida tropicalis*	Wild-type	[34]
AM2004/0069	*Candida tropicalis*	Wild-type	[34]
SC5314	*Candida albicans*	Wild-type	[35]
ATCC 6260	*Candida guilliermondii*	Wild-type	[36]
ATCC 6258	*Candida krusei*	Wild-type	ATCC
SZMC 8110	*Candida parapsilosis*	Wild-type	[37]
SZMC 1545	*Candida orthopsilosis*	Wild-type	[37]
SZMC 1548	*Candida metapsilosis*	Wild-type	[37]
VPCI 479/P/13	*Candida auris*	Wild-type	[38]
